# Genetic Transsynaptic Techniques for Mapping Neural Circuits in *Drosophila*

**DOI:** 10.3389/fncir.2021.749586

**Published:** 2021-10-04

**Authors:** Lina Ni

**Affiliations:** School of Neuroscience, Virginia Tech, Blacksburg, VA, United States

**Keywords:** *Drosophila*, neural circuits, transsynaptic labeling, *trans*-Tango, *TRACT*, BAcTrace

## Abstract

A neural circuit is composed of a population of neurons that are interconnected by synapses and carry out a specific function when activated. It is the structural framework for all brain functions. Its impairments often cause diseases in the nervous system. To understand computations and functions in a brain circuit, it is of crucial importance to identify how neurons in this circuit are connected. Genetic transsynaptic techniques provide opportunities to efficiently answer this question. These techniques label synapses or across synapses to unbiasedly label synaptic partners. They allow for mapping neural circuits with high reproducibility and throughput, as well as provide genetic access to synaptically connected neurons that enables visualization and manipulation of these neurons simultaneously. This review focuses on three recently developed *Drosophila* genetic transsynaptic tools for detecting chemical synapses, highlights their advantages and potential pitfalls, and discusses the future development needs of these techniques.

## Introduction

A neural circuit is composed of a population of neurons that are interconnected by synapses and carry out a specific function when activated (Purves et al., 2011). It is the structural framework for all brain functions, such as processing perception and cognition and coordinating behavior. Its impairments often cause diseases in the nervous system (Rubenstein and Merzenich, 2003; Belmonte et al., 2004; Lynall et al., 2010). To understand computations and functions in a brain circuit, it is important to identify how neurons in this circuit are connected.

*Drosophila melanogaster* is an attractive model to study the circuit basis of animal behavior. *Drosophila* has extensive collections of genetic reagents that can be used to label and manipulate most cell types, including different classes of neurons. On the other hand, *Drosophila* has a relatively small nervous system, ∼100,000 neurons in an adult fly brain, controlling various sophisticated behaviors. The simplicity of the neural system and rich genetic reagents provide feasibility to understand how neural circuits connect and control behaviors (Venken et al., 2011).

There has been rapid development in techniques for mapping *Drosophila* neural circuits. Electron microscopy (EM) and paired electrophysiology recordings are two golden standards for unambiguously mapping synaptic connectivity, but both are labor-intensive and time-consuming. The *Drosophila* connectome has been created by EM and provides information on all circuits of the central brain (Scheffer et al., 2020). However, it is impractical to apply EM to analyze multiple samples, let alone high-throughput screens. Other methods, such as labeling pre- and postsynaptic proteins by immunohistochemistry or fluorescent reporters, allow for analysis of neural circuits by light microscopy. These methods detect the proximity of two markers rather than synapses because synaptic contacts cannot be resolved by regular light microscopy. Although super-resolution light microscopy could detect synaptic structures, they usually have rigorous requirements for the instrument and sample preparation (Carvalhais et al., 2020). Activity-dependent methods, such as optogenetics and calcium imaging, enable to identify circuit connections through functional analysis. These methods are suited for confirming, not discovering, synaptic contacts since both pre- and postsynaptic neurons that form the synapse must be known and have driver lines. These techniques have been well-reviewed elsewhere and will not be discussed in this review (Meinertzhagen and Lee, 2012; Lee et al., 2017; Luo et al., 2018; Guo et al., 2019).

A class of genetic tools that label synapses or across synapses to label synaptic partners are referred to as genetic transsynaptic tools. Tools to label postsynaptic neurons are termed anterograde, while retrograde tools reveal presynaptic neurons. GRASP (GFP Reconstitution Across Synaptic Partner) is a well-established genetic transsynaptic tool that was initially developed in *Caenorhabditis elegans* and has been used to identify synaptic contacts in various genetic model organisms, including *Drosophila* (Feinberg et al., 2008). It labels synapses based on the proximity of pre- and postsynaptic plasma membranes and allows for visualization of synaptic connection between two neurons by light microscopy. GRASP contains two split-GFP fragments, spGFP1-10 and spGFP11, that are extracellularly expressed in presynaptic and postsynaptic neurons. While neither fragment fluoresces individually, GFP is reconstituted transsynaptically and exhibits fluorescence when two neuron populations connect. The original *Drosophila* GRASP fuses both split-GFP fragments to the extracellular domain of the CD4 transmembrane protein (Gordon and Scott, 2009). This version is not synaptically targeted and potentially leads to false-positive signals at non-synaptic locations. To avoid non-synaptic false-positive signals, enhanced variants of GRASP have been developed, in which one or both components of GRASP are targeted to synapses, thereby restricting GFP reconstitution to synapses (Fan et al., 2013; Shearin et al., 2018). Moreover, an activity-dependent, multi-color GRASP is synthesized by fusing the spGFP1-10 fragment, or its variants, to the C terminus of *Drosophila* neuronal-synaptobrevin, which can be used to distinguish active from inactive synapses (Macpherson et al., 2015).

Besides chemical synapses, electrical synapses, formed by gap junctions, also contribute to brain functions. Electrical synaptic partners could be detected by PARIS (Pairing Actuators and Receivers to optically ISolate gap junctions) (Wu et al., 2019). In PARIS, actuator and receiver cells express the light-gated proton pump ArchT and pH-sensitive fluorescent protein pHluorin, respectively. When ArchT is activated, it pumps hydrogen out of actuator cells. If actuator and receiver cells connect through gap junctions, pHluorin responds to the change of hydrogen and so its fluorescence increases. PARIS not only detects gap junctions but also resolves their subcellular locations.

This review focuses on technical advances of three recently developed genetic transsynaptic tools for detecting chemical synapses: *trans*-Tango (Talay et al., 2017), TRACT (TRAnsneuronal Control of Transcription) (Huang et al., 2017), and BAcTrace (Botulinum-Activated Tracer) (Cachero et al., 2020). Unlike GRASP that labels synapses, these methods label and provide genetic access to synaptic partners. In this review, we will highlight advantages and potential pitfalls of these techniques, as well as discuss their future development needs.

## Genetic Transsynaptic Tools

**FIGURE 1:**
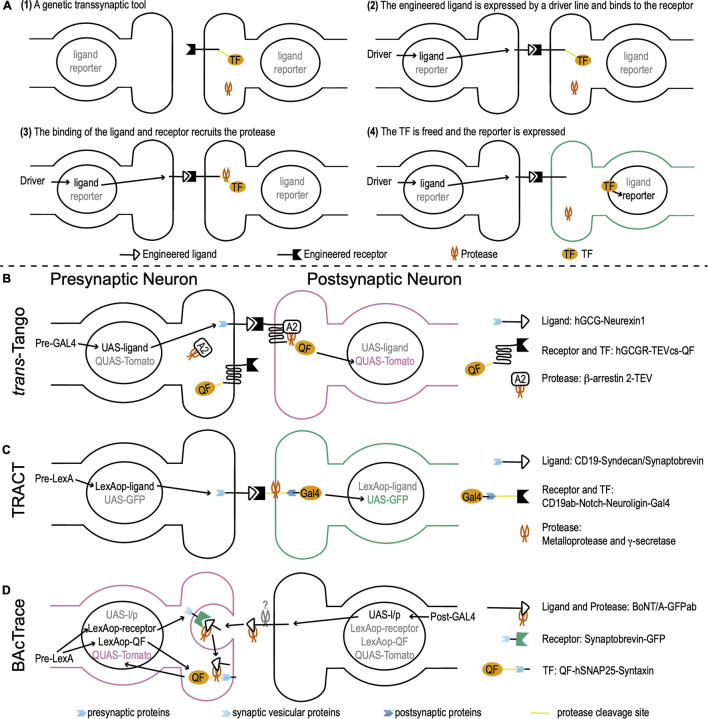
*Drosophila* genetic transsynaptic techniques. **(A)** Schematic diagram depicting the principle and process of genetic transsynaptic tools. (1) A genetic transsynaptic tool often includes five components: an engineered receptor, an engineered ligand, a protease, a transcription factor (TF), and a reporter. (2) When a genetic transsynaptic tool is applied, a driver line for neurons of interest drives the expression of the engineered ligand. (3) The ligand binds to the engineered receptor on synaptically connected neurons and recruits the protease. (4) TF is freed and translocates to nuclei, where it activates the expression of the reporter to label synaptic partners. **(B)** *trans-*Tango. The receptor is hGCGR. The ligand is hGCG. The ligand is linked to *Drosophila* neurexin 1 (blue feather) for the presynaptic localization. The protease is TEV. It is tethered to β-arrestin 2 (A2) (β-arrestin 2-TEV). The TF is QF. QF is tethered to the C terminus of receptor by a cleavage site of TEV (TEVcs) (yellow line). The reporter is Tomato that is controlled by QUAS (*QUAS-Tomato*). The receptor, TF, and protease are panneuronally expressed (the DNA information is not shown), while the ligand is controlled by UAS (*UAS-ligand*). Activation of *trans-*Tango requires a presynaptic GAL4 (*Pre-GAL4*). **(C)** TRAnsneuronal Control of Transcription (TRACT). The receptor includes a CD19 antibody (CD19ab), the Notch regulatory region and transmembrane domain (yellow line), and the cytosolic domain of *Drosophila* neuroligin (dark blue feather) for postsynaptic localization. The ligand is CD19, whose C terminus is linked to the cytosolic domain of either syndecan or synaptobrevin (blue feather) for presynaptic localization (CD19-Syndecan/Synaptobrevin). The proteases are ubiquitously present metalloprotease and γ-secretase (the DNA information is not shown). They cleave the receptor at the Notch regulatory region and transmembrane domain. The transcription factor (TF) is GAL4 that is tethered to the C terminus of the receptor. The reporter is GFP controlled by UAS (*UAS-GFP*). The receptor and TF are panneuronally expressed (the DNA information is not shown; this fusion protein is linked to a postsynaptic protein and thus is not shown in the presynaptic terminal) and the ligand is controlled by LexAop (*LexAop-ligand*). Activation of TRACT requires a presynaptic LexA (*Pre-LexA*). **(D)** BAcTrace. The receptor is a fusion protein, in which a GFP is linked with *Drosophila* synaptobrevin, a vesicular protein in presynaptic terminals (light blue feather) (Synaptobrevin-GFP). The ligand is engineered from BoNT/A, whose receptor-binding domain is replaced by a GFP antibody (GFPab). The protease is the light chain of BoNT/A. The TF is QF. QF is linked with hSNAP25 (yellow line), the cleavage site of the light chain protease. This fusion protein is targeted to the presynaptic membrane by *Drosophila* presynaptic syntaxin (blue feather) (QF-hSNAP25-Syntaxin). The reporter is Tomato controlled by QUAS (*QUAS-Tomato*). The ligand/protease is controlled by UAS (*UAS-l/p*), while the receptor and TF are controlled by LexAop (*LexAop-receptor* and *LexAop-QF*). Activation of BAcTrace requires a postsynaptic GAL4 (*Post-GAL4*) and a presynaptic LexA (*Pre-LexA*).

Genetic transsynaptic tools that label synaptic partners include five components: engineered receptor, engineered ligand, protease, transcription factor (TF), and reporter (Figure 1A). The engineered receptor and ligand are fusion proteins. Both contain interactive domains that bind to each other, but not to any molecules that exist in wild-type flies. To avoid non-synaptic signals, the ligand and/or receptor contain synaptic proteins that target them to pre- or postsynaptic membranes. The proximity between pre- and postsynaptic membranes enables the binding between the ligand and receptor. The TF is exogenous whose DNA binding sequence does not present in wild-type flies. It is usually sequestered to the plasma membrane and doesn’t move to nuclei. The reporter is a fluorescent protein that is controlled by the activation DNA sequence of the TF. When a genetic transsynaptic tool is applied, a driver line for neurons of interest drives the expression of the engineered ligand. The ligand binds to the engineered receptor on synaptically connected neurons across synaptic clefts (Figure 1A2). This binding recruits the protease, which in turn frees the TF (Figure 1A3). The TF then translocates to nuclei, binds to its activation DNA sequence, and activates the expression of the reporter to label synaptic partners (Figure 1A4).

### *trans*-Tango

The design of *trans-*Tango is based on the Tango assay that transforms transient interaction between G protein-coupled receptors and their ligands to a more stable readout (Barnea et al., 2008; Talay et al., 2017). The receptor in *trans-*Tango is the human glucagon G protein-coupled receptor (hGCGR). The ligand is a mutated version of the glucagon peptide that has high potency for hGCGR activation (hGCG). hGCG is tethered to *Drosophila* neurexin 1 that localizes hGCG to the presynaptic membrane. The protease is an N1a protease from the tobacco etch virus TEV (TEVcs). TEV is linked to human β-arrestin 2, which is recruited to the receptor when the receptor is bound to and activated by the ligand hGCG. The TF is a fungal transcription factor QF. QF is tethered to the C terminus of the receptor hGCGR by a cleavage site of TEV. The reporter is a fluorescent protein Tomato that is controlled by a QF activation sequence QUAS. The receptor, TF, and protease are panneuronally expressed, while the ligand is controlled by a UAS sequence. UAS is the activation DNA sequence of a yeast TF GAL4. When a presynaptic GAL4 is expressed, it drives the expression of the ligand hGCG in presynaptic neurons. hGCG binds to and activates the receptor hGCGR on the postsynaptic membrane. Activated hGCGR, in turn, recruits β-arrestin 2 that links to the protease TEV. TEV cleaves the link between hGCGR and QF to free QF. QF then translocates to nuclei and drives the expression of the reporter Tomato in postsynaptic neurons (Figure 1B).

### TRACT

TRACT adopts the molecular mechanism of the Notch signaling pathway to monitor and modify postsynaptic neurons (Huang et al., 2017). The receptor is engineered from Notch and maintains its regulatory region and transmembrane domain. The extracellular domain is replaced by a CD19 antibody (CD19ab). The C terminus is linked to the cytosolic domain of *Drosophila* neuroligin for postsynaptic localization. The ligand is the mouse lymphocyte antigen CD19. Its C terminus is linked to the cytosolic domain of either syndecan or synaptobrevin for presynaptic localization. The TF is a simplified version of GAL4 that is tethered to the C terminus of the receptor. Two ubiquitously present proteases (metalloprotease and γ-secretase) cleave the Notch regulatory region and transmembrane domain to free GAL4. The reporter is a fluorescent protein GFP controlled by a UAS sequence. The receptor and TF are panneuronally expressed and the ligand is controlled by a LexAop sequence. The LexAop sequence is the activation DNA sequence of a bacterial TF LexA. When a presynaptic LexA is expressed, it drives the expression of ligands in presynaptic terminals. CD19 binds to CD19ab and activates the receptor to free GAL4. GAL4 then translocates to nuclei and drives the expression of the reporter GFP in postsynaptic neurons (Figure 1C).

### BAcTrace

Unlike *trans-*Tango and TRACT that trace anterograde circuits from presynaptic neurons to postsynaptic neurons, BAcTrace is designed for retrograde tracing (Cachero et al., 2020). While both *trans-*Tango and TRACT are contact-based systems, labeling in the BAcTrace system is triggered by protein transfer between connected neurons. BAcTrace adopts the molecular mechanism of *Clostridium botulinum* neurotoxin A1 (BoNT/A). BoNT/A contains a light chain and a heavy chain. The light chain is a highly specific protease for human SNAP25 (hSNAP25) but does not cleave *Drosophila* SNAP25. With the assistant of the heavy chain, the light chain gets into the presynaptic terminal from the synaptic cleft during synaptic vesicle recycling.

The receptor in BAcTrace is a fusion protein, in which a GFP is linked with synaptobrevin, a vesicular protein in presynaptic terminals. The ligand is engineered from BoNT/A, whose receptor-binding domain is replaced by a GFP antibody (GFPab). The protease is the light chain of BoNT/A. The TF is QF. QF is linked with hSNAP25 and this fusion protein is targeted to the presynaptic membrane by *Drosophila* presynaptic syntaxin. The reporter is Tomato controlled by QUAS. The ligand/protease is controlled by UAS, while the receptor and TF are controlled by LexAop. When a postsynaptic GAL4 is expressed, it drives the expression of the ligand/protease in postsynaptic neurons. At the same time, a presynaptic LexA is expressed and drives the expression of the receptor and TF in presynaptic neurons. The ligand is released from the postsynaptic membrane by an unknown mechanism and binds to the receptor. This binding guides the BoNT/A light chain protease to get into the presynaptic terminal during synaptic vesicle recycling. Then, the BoNT/A light chain cleaves hSNAP25 and frees QF. QF translocates to nuclei and drives the expression of Tomato in presynaptic neurons (Figure 1D).

These techniques, as well as GRASP and PARIS, are compared in Table 1 to help potential users to select suitable tools according to their experimental goals and available driver lines.

**TABLE 1:** Comparison of genetic transsynaptic tools.

	GRASP	*trans-*Tango	TRACT	BAcTrace	PARIS
Label chemical or electrical synapses	Chemical	Chemical	Chemical	Chemical	Electrical
Anterograde or retrograde tracing	Both	Anterograde	Anterograde	Retrograde	Both
Label synapses or synaptic partner cells	Synapses	Partner cells	Partner cells	Partner cells	Partner cells
Detect synaptic activity	Yes	No	No	No	No
Label multiple synapses/synaptic partners by different reporters	Yes	No	No	No	No
Access to synaptic partner cells without driver lines	No	Yes	Yes	Yes	No
Required driver lines	Pre & post*	Pre-Gal4	Pre-LexA	Post-Gal4^$^	Pre and post*
Known toxicity	No	No	No	Yes	No

*The pre- and postsynaptic driver lines must be from different binary transcription systems and have no overlap expression.

*^$^The transcription factor (TF) construct in BAcTrace, QF-hSNAP25-Syntaxin, is toxic and cannot be panneuronally expressed, thus a driver line is required to express it. This driver line is not necessary to be specific to presynaptic partners of neurons of interest but could label a relatively broad group of neurons. BAcTrace “picks” and labels presynaptic neurons. Of note, the expression of this driver line cannot overlap with the postsynaptic Gal4 line.*

## Applications of Genetic Transsynaptic Tools

Although these genetic transsynaptic techniques are new, they have been widely used to label synaptic partners in various *Drosophila* neural circuits. For example, since it was developed in 2017, *trans-*Tango has been used to identify postsynaptic neurons in visual circuits (Zhao et al., 2019; Keleş et al., 2020; Kiral et al., 2020; Hardcastle et al., 2021; Song et al., 2021), auditory circuits (Kim et al., 2020), olfactory circuits (Talay et al., 2017; Lerner et al., 2020), taste circuits (Talay et al., 2017; Chen et al., 2019), mechanosensory circuits (Suver et al., 2019), motor circuits (Feng et al., 2020; Yalgin et al., 2020; Buhl et al., 2021), courtship circuits (He et al., 2019; Liu et al., 2020), learning and memory circuits (Scaplen et al., 2020; Georganta et al., 2021), and circuits controlling aggression (Hu et al., 2020; Wu et al., 2020), social attraction (Sun et al., 2020), and circadian rhythm and sleep (Guo et al., 2018; Dreyer et al., 2019; Liu et al., 2019; Duhart et al., 2020). It has also been applied to identify neuropeptide synapses (Zandawala et al., 2018, 2021) and other neuronal targets, such as the adipose tissue (Scopelliti et al., 2019). In addition, *trans-*Tango has been adopted to discover postsynaptic neurons in the central brain (Omoto et al., 2018; Scaplen et al., 2021). The expression of *trans-*Tango in mutant backgrounds helps understand the function of mutant genes in brain wiring (Chen et al., 2019; Kiral et al., 2020). Moreover, optogenetic/chemogenetic and calcium imaging techniques have been combined with *trans-*Tango to understand the functional connectivity between pre- and postsynaptic neurons (Guo et al., 2018; He et al., 2019; Feng et al., 2020). TRACT has been used to label postsynaptic neurons in olfactory and rhythm circuits (Huang et al., 2017), as well as glial cells (Yin et al., 2021). BAcTrace has been applied to show synaptic connections from olfactory projection neurons to olfactory receptor neurons and Kenyon cells of mushroom bodies and lateral horn neurons (Cachero et al., 2020).

## Advantages of Genetic Transsynaptic Tools

Genetic transsynaptic techniques have several advantages. First, *trans-*Tango, TRACT, and BAcTrace enable the discovery of candidate synaptic partners using light microscopy. In principle, these techniques can be applied to any neural circuit because neurons of interest form direct synaptic connections with labeled neurons in an unbiased manner. They can also be combined with mosaic or intersectional approaches for sparse labeling of synaptic partners, which renders them suitable for tracing projections within dense neuropil. Moreover, in *trans-*Tango and BAcTrace, the labeling of synaptic partners is age-dependent—older flies show stronger labeling. This age dependence may correlate with the strength of synaptic connections and thus these techniques could be used to characterize the strength of a connection under different conditions.

Second, these techniques generate highly reproducible transsynaptic labeling results and can be used in high-throughput experiments given that they are not labor-intensive or time-consuming. They also combine user-friendly genetics for direct application. For example, *trans-*Tango needs a single cross to any GAL4 line of interest to detect their postsynaptic neurons. Hereafter, genetic transsynaptic techniques would be used to perform screens to identify changes in neuronal connectivity due to genetic mutations or in response to environmental chemicals, behavioral experiences, or diseases.

Third, genetic transsynaptic techniques allow for efficient visualization and genetic manipulation of synaptic partners simultaneously. These techniques provide genetic access to synaptic partners in living animals. For this reason, they can be used to monitor the activity of synaptically connected neurons by expressing genetically encoded Ca^2+^ sensors, or to manipulate their activity by expressing optogenetic tools. They can also be used to regulate the gene expression in synaptic partners. Combined with appropriate behavioral assays, these systems enable the establishment of novel neural connectivity and behavioral causality.

These advantages make genetic transsynaptic techniques valuable for mapping neural circuits despite the fly connectome has been created by EM.

## Limitations of Genetic Transsynaptic Tools

While genetic transsynaptic techniques have many benefits, they still should be applied and interpreted with caution. (1) The synaptic strength affects the transsynaptic labeling. Labeling of weak synapses requires a higher level of the receptor and/or ligand expression. (2) These techniques require driver lines of neurons of interest. BAcTrace even requires driver lines for both pre- and postsynaptic neurons and their expression cannot overlap. Moreover, the strength of driver lines affects the accuracy of the transsynaptic labeling. (3) These techniques require the reconstitution of an exogenous cell-to-cell signaling apparatus, which adds genetic complexity and may cause toxicity. For example, the TF construct QF-hSNAP25-Syntaxin in BAcTrace is toxic and cannot be panneuronally expressed. (4) Ligands and/or receptors are targeted to synapses using different pre- or postsynaptic proteins. These proteins might have slightly different locations at pre- or postsynaptic sites. They may also influence the abundance and/or stability of ligands and receptors. (5) They all have false-positive and/or negative issues. False-positive signals may be due to the overexpression artifacts if the receptor and/or ligand molecules escape synaptic confinement. False-negative issues may be due to the low level of the receptor and/or ligand expression or the inconsistency of driver lines. Therefore, candidate synaptic partners identified by genetic transsynaptic techniques require validation using complementary methods, such as paired recordings and EM. Paired recordings examine functional connections between two neurons, while EM identifies the synaptic structure. The fly connectome provides free online tools to match EM and light microscopy data, which greatly shortens the time for exclusion of false-positive or negative signals.

## Future Development Needs

Genetic transsynaptic techniques provide genetic access to synaptic partners and have been widely applied for mapping neural circuits in flies. In the future, these tools are expected to provide user-friendly genetics for following functions. (1) Label individual synaptic partners. A neuron usually has more than one synaptic partner. To examine the function of each synaptic partner, they must be individually labeled. It is important to develop a general strategy to sparsely label synaptic partners in different neural circuits. (2) Monitor the activity of labeled synaptic partners. To achieve this goal, genetically encoded calcium indicators or voltage sensors need to be expressed in synaptic partners. (3) Activate or inactivate labeled synaptic partners to understand their functions. Optogenetic or other genetic components can be expressed in labeled synaptic partners to manipulate their activities. The causal basis of a corresponding behavior can be examined. (4) Manipulate the gene expression in labeled synaptic partners. Overexpression or knockdown of a gene can be achieved by using the corresponding TF to express the gene or its shRNA. (5) Label higher-order neurons. In principle, the combined use of TRACT and *trans-*Tango allows labeling third-order neurons. Combining *QUAS-Gal4* with *trans-*Tango and BAcTrace or *UAS-LexA* with TRACT may target ligands in synaptic partners and, consequently, label the whole neural circuits. (6) Retrograde labeling by *trans-*Tango and TRACT. Both techniques have the potential to trace retrograde circuits by the postsynaptic expression of ligands and the presynaptic expression of receptors.

In principle, genetic transsynaptic techniques can be used in any organism amenable to transgenesis and allow for genetic access to cells based on their connectivity. These techniques will benefit the study of neurological disorders and the development of effective treatments.

## Author Contributions

LN drafted the initial manuscript and approved the final version of the manuscript.

## Conflict of Interest

The author declares that the research was conducted in the absence of any commercial or financial relationships that could be construed as a potential conflict of interest.

## Publisher’s Note

All claims expressed in this article are solely those of the authors and do not necessarily represent those of their affiliated organizations, or those of the publisher, the editors and the reviewers. Any product that may be evaluated in this article, or claim that may be made by its manufacturer, is not guaranteed or endorsed by the publisher.
